# The Toronto Mindfulness Scale and the State Mindfulness Scale: psychometric properties of the Spanish versions

**DOI:** 10.3389/fpsyg.2023.1212036

**Published:** 2023-07-06

**Authors:** Jaime Navarrete, Marta Fontana-McNally, Ariadna Colomer-Carbonell, Juan P. Sanabria-Mazo, Daniel Pinazo, Antonio-José Silvestre-López, Mark Lau, Galia Tanay, Javier García-Campayo, Marcelo Demarzo, Joaquim Soler, Ausiàs Cebolla, Albert Feliu-Soler, Juan V. Luciano

**Affiliations:** ^1^Institut De Recerca Sant Joan De Déu, Esplugues De Llobregat, Spain; ^2^Teaching, Research & Innovation Unit, Parc Sanitari Sant Joan De Déu, Sant Boi De Llobregat, Spain; ^3^CIBER of Epidemiology and Public Health (CIBERESP), Madrid, Spain; ^4^Department of Evolutionary, Educational and Social Psychology and Methodology, Universitat Jaume I, Castellón de la Plana, Spain; ^5^Department of English Studies, Universitat Jaume I, Castellón de la Plana, Spain; ^6^Department of Psychiatry, Faculty of Medicine, University of British Columbia, Vancouver, BC, Canada; ^7^Observing Minds Lab, School of Psychological Sciences, University of Haifa, Haifa, Israel; ^8^Institute of Health Research of Aragon (IIS Aragón), Miguel Servet University Hospital, Zaragoza, Spain; ^9^Universidad de Zaragoza, Zaragoza, Spain; ^10^Mente Aberta - the Brazilian Center for Mindfulness and Health Promotion- Universidade Federal de São Paulo- UNIFESP, São Paulo, Brazil; ^11^Department of Psychiatry, Hospital de la Santa Creu i Sant Pau, Barcelona, Spain; ^12^CIBER of Mental Health (CIBERSAM), Madrid, Spain; ^13^Department of Personality, Evaluation and Psychological Treatments, University of Valencia, Valencia, Spain; ^14^CIBER of Obesity and Nutrition (CIBEROBN), Madrid, Spain; ^15^Department of Clinical & Health Psychology, Autonomous University of Barcelona, Bellaterra, Spain

**Keywords:** toronto mindfulness scale, state mindfulness scale, spanish validation, confirmatory factor analysis, WLSMV, omega-hierarchical

## Abstract

**Objectives:**

The Toronto Mindfulness Scale (TMS) and the State Mindfulness Scale (SMS) are two relevant self-report measures of state mindfulness. The purpose of this study was to examine the internal structure and to offer evidence of the reliability and validity of the Spanish versions of the TMS and SMS.

**Methods:**

Data from six distinct non-clinical samples in Spain were obtained. They responded to the TMS (*n* = 119), SMS (*n* = 223), and measures of trait mindfulness, decentering, non-attachment, depression, anxiety, stress, positive and negative affect, self-criticism, and self-reassurance. The internal structure of the TMS and SMS was analyzed through confirmatory factor analysis. Reliability, construct validity, and sensitivity to change analyses were performed.

**Results:**

The correlated two-factor structure (curiosity and decentering) was the best-fitting model for the TMS (CFI = 0.932; TLI = 0.913; RMSEA = 0.100 [0.077–0.123]; WRMR = 0.908). The bifactor structure (general factor, mindfulness of body, and mindfulness of mind) was the best-fitting model for the SMS (CFI = 0.961; TLI = 0.950; RMSEA = 0.096 [0.086–0.106]; WRMR = 0.993). Adequate reliability was found for both measures. The reliability of the SMS specific factors was very poor when controlling for the general factor. The patterns of correlations were mainly as expected and according to previous literature. The TMS and SMS have been able to detect state mindfulness changes after different meditation practices.

**Conclusion:**

Validity evidence is provided to support the use of the TMS and SMS in Spanish populations, though the reliability of the SMS specific factors merit revision.

## 1. Introduction

Mindfulness is generally defined as a non-elaborative, non-judgmental, present-centered awareness where different thoughts, feelings, and sensations that arise in one’s attentional field are acknowledged and accepted as they are (Kabat-Zinn, 1990). It has been described as a trait-like kind of awareness, though mindfulness can also be viewed as a mode, or state-like quality, that is maintained only when attention to experience is intentionally set with an open, non-judgmental orientation to experience (Bishop et al., 2004). In fact, the rationale of mindfulness-based interventions (MBI) is that evoking the state of mindfulness regularly across meditation practice increases the propensity of an individual toward mindfulness in everyday life (Davidson, 2010). In this regard, Kiken et al. (2015) showed that repeated mindfulness meditation practice within an MBI increased individuals’ state mindfulness over time, which in turn, predicted changes in trait mindfulness and psychological distress.

As repeated measures of key state variables during MBIs contribute to a better understanding of the trajectory of mindfulness training (Navarrete et al., 2022), the need for researchers and clinicians to have access to reliable measures is growing. Nevertheless, when attempting to measure mindfulness, the difficulty arises depending on whether trait or state mindfulness is the target. Most self-report measures of mindfulness assess the construct as a trait-like behavior (i.e., a general tendency to be mindful in daily life) as opposed to a state-like construct (Siegling and Petrides, 2014; Baer, 2019). An example of the most popular trait mindfulness measure is the Five-Facet Mindfulness Questionnaire (FFMQ; Baer et al., 2006), which is used worldwide (Lecuona et al., 2020). In contrast, there are very few self-report measures focused on state mindfulness, which consequently means that their psychometric properties have been less studied (Baer, 2019).

Currently, the two most important self-report measures of state mindfulness are (Baer, 2019): the Toronto Mindfulness Scale (TMS; Lau et al., 2006) and the State Mindfulness Scale (SMS; Tanay and Bernstein, 2013). The TMS is a reliable and valid measure of state mindfulness, derived from Bishop et al.’s (2004) operational definition of mindfulness which comprised the components of ‘Orientation to Experience’ -characterized by openness and curiosity- and ‘Self-Regulation of Attention’ – being aware of current thoughts, feelings, and sensations without getting caught up in ruminative or elaborative thinking-. In contrast, the original SMS framework was based on an integration of the traditional Buddhist concept of mindfulness and also Bishop et al.’s (2004) construct definition.

The TMS is a 13-item scale that encompasses two separate factors: curiosity and decentering. Curiosity refers to the quality with which one becomes aware in the present moment, while decentering refers to the ability to be aware of one’s experience without being drawn in by the different stimuli (Lau et al., 2006). The TMS has been adapted and validated to Korean (Lee et al., 2010) and Chinese (Yu et al., 2021) populations, though only the last performed factor analysis. In addition, Ireland et al. (2019) replicated and expanded Lau et al. (2006) findings regarding the original –English- version of the TMS. Regarding dimensionality, Ireland et al. (2019) and Yu et al. (2021) found support for the two-factor structure after implementing some modifications that mainly involved the curiosity factor. Overall, the TMS showed a good internal consistency and adequate convergent/discriminant validity, correlating positively with trait mindfulness (only decentering factor), decentering, frequency of meditation practice, wellbeing, self-awareness, inner peace, positive affect, and negatively with stress, depression, and anxiety with a small-to-medium strength for all the associations (*r* range from 0.02 to 0.54; Lee et al., 2010; Ireland et al., 2019; Yu et al., 2021).

The SMS is a 21-item scale that assesses two state mindfulness aspects: one reflecting state mindfulness of bodily sensations and the other reflecting state mindfulness of mind. Dimensionality analyses from the original validation study showed that the SMS entailed a hierarchical two-factor structure including a higher-order state mindfulness factor (Tanay and Bernstein, 2013). As the original authors noted (Ruimi et al., 2022), this scale has been translated in to various languages, including Spanish, and has been used in many research settings (e.g., Navarrete et al., 2021a). However, except for the original work by Tanay and Bernstein (2013), only one study has assessed its psychometric properties in another culture (Andrade et al., 2019). In a Portuguese convenience sample, Andrade et al. (2019) showed that the original hierarchical two-factor structure plus four pairs of correlated error terms were not supported. In addition, they tested a modified hierarchical two-factor structure with six pairs of correlated error terms, for which they found an adequate goodness of fit. In both Tanay and Bernstein (2013) and Andrade et al. (2019) studies, internal consistency was adequate and convergent/discriminant validity analyses were in the expected direction, showing that state mindfulness was positively correlated with curiosity, decentering, some facets of trait mindfulness, and positive affect, and negatively with suppression emotion regulation strategy with a small-to-medium strength for all the associations (*r* range from 0.05 to 0.56; Tanay and Bernstein, 2013; Andrade et al., 2019).

As mindfulness research grows among Spanish-speaking countries, the need for reliable and valid Spanish adaptations of these scales arises (Hervás et al., 2016). Moreover, further psychometric studies of the TMS and SMS are warranted in order to clarify some aspects about their factor structure (Ireland et al., 2019; Ruimi et al., 2022). Finally, previous studies used maximum likelihood or maximum likelihood robust estimation methods when confirmatory factor analyses were conducted, which are less optimal methods for Likert-type data than diagonally weighted least squares (Li, 2016).

Against this background, the present work assesses the psychometric properties of the Spanish versions of the TMS and the SMS in pooled samples from the Spanish general population. More specifically, the objectives of this study were to analyze the factor structure of both scales (i.e., the TMS and the SMS), their reliability, convergent/discriminant validity, and sensitivity to change. Regarding the first objective, we expected that a correlated two-factor model and a hierarchical two-factor model for the TMS and the SMS, respectively, would yield the best fit in our dataset (Hypothesis 1). Moreover, we expected adequate internal reliability (Hypothesis 2) and convergent/discriminant validity for these measures. In this sense, we hypothesized that the TMS scores would be positively associated with mindfulness facets, decentering, and non-attachment, as well as negatively associated with depression, anxiety, and stress with a small-to-medium strength for all the associations (Hypothesis 3). Similarly, we hypothesized that SMS scores would be positively associated with TMS scores (strong association expected), trait mindfulness facets (i.e., observing, describe, non-judging, and non-reactivity), positive affect, and self-reassurance, as well as negatively associated with self-criticism with a small-to-medium strength for all the associations (Hypothesis 4). Finally, it was anticipated that both state mindfulness measures would be sensitive to change after meditation practice, showing statistically significant higher TMS and SMS scores in participants after common meditation practices (Hypothesis 5).

## 2. Materials and methods

### 2.1. Participants

The dataset for this study stemmed from six non-clinical samples from the Spanish general population. Characteristics of each sample are shown in Table 1. Overall, participants were primarily women and university students. Sample 1 comprised a convenience sample of undergraduates from the University of Jaume I (Castellón, Spain). Sample 2 comprised individuals with previous meditation experience from the University of Zaragoza community (Zaragoza, Spain). Sample 3 comprised participants that took part in an efficacy study of a brief mindful eating induction on food choices and intake at the Basque Culinary Center in Spain (Allirot et al., 2018). Participants in Sample 4 were recruited from the University of Zaragoza for the psychometric study on the Compassion Practice Quality Scale (Navarrete et al., 2021b). Sample 5 was composed of subjects from an ongoing study about compassion practice quality at the University of Valencia (Valencia, Spain). Finally, Sample 6 comprised a convenience sample of students and administration and services staff from the University of Jaume I (Castellón, Spain).

**Table 1:** Participants characteristics for included samples.

	TMS		SMS			TMS and SMS
Characteristics	Sample 1 (*n* = 119)	Sample 2 (*n* = 24)	Sample 3 (*n* = 70)	Sample 4 (*n* = 69)	Sample 5 (*n* = 84)	Sample 6 (*n* = 42)
Gender (women): *n* (%)	92 (77.3)	18 (75)	70 (100)	50 (72.50)	65 (77.40)	26 (61.90)
Age (in years): *M (SD)*	20.36 (3.13)	52.59 (8.32)	35.30 (10.68)	35.77 (11.29)	31.43 (12.87)	33.81 (9.77)
Previous experience in meditation practice (yes): *n* (%)	27 (22.7)	24 (100)	0 (0)	32 (46.40)	37 (44)	13 (31)
Level of education: *n* (%)						
Some school education	0 (0)	0 (0)	n/a	8 (11.60)	1 (1.20)	0 (0)
High school and/or vocational education	0 (0)	0 (0)	n/a	7 (10.10)	7 (8.30)	8 (19)
University degree/professional qualification	119 (100)	24 (100)	n/a	54 (78.30)	76 (90.50)	34 (81)

### 2.2. Procedure

Initially, permission from the original authors was obtained for translating and validating the TMS and SMS. A team of Spanish psychologists, who were proficient in English and experts in mindfulness interventions and contemplative psychology, translated the original version of the TMS and SMS into Spanish. Then, discrepancies were discussed and the items were back-translated into English by a native English speaker also fluent in Spanish and independent from the team. Again, discrepancies with the original TMS and SMS were discussed and the Spanish versions were adapted until they were equivalent to the English versions. The final versions of the Spanish TMS and SMS can be found in the supplemental materials section (Supplementary Appendix A).

This psychometric study was approved by the Ethics Committee at the Sant Joan de Déu Foundation (PIC-141-19). Participants from all samples voluntarily gave their written informed consent to take part in their respective study and gave permission to analyze their data in any subsequent study. All studies complied with the Declaration of Helsinki and no remuneration was offered for participating in any of them. Data from all samples were obtained from different research projects described below. The main researchers of each project provided data that has not been previously analyzed or published in any scientific journal.

Sample 1 participants were from a research project about the association between meditation practice and values-related behaviors. The main aim was to examine the processes involved in that association. A cross-sectional design was used. Participants were recruited mainly through advertisements in several Spanish websites about mindfulness, meditation, and psychology (scientific associations, mindfulness associations, monasteries, etc.), as well as on non-professional social networks (i.e., Facebook). Participants completed an online assessment protocol. Complementary information about this research can be found in its main publication (Franquesa et al., 2017).

Sample 2 participants underwent a 1-month Vipassana meditation retreat organized by a master’s degree course in mindfulness in the University of Zaragoza (Spain). Individuals who had confirmed their presence at the retreat were sent a letter inviting them to participate in a longitudinal study aimed to assess changes in mindfulness, well-being, and prosocial personality traits. Participants answered different measures in the paper-and-pencil format. Concretely, the TMS was administered immediately before the retreat opening and at the end of the retreat. During the retreat, participants practiced open monitoring and focused attention meditations (8–9 h of daily meditative practice) and had 1–2 h of teachings. Complementary information about this study can be found in its main publication (Montero-Marin et al., 2016).

Sample 3 participants were women from a longitudinal study about the effect of a single mindful eating induction on subsequent food choices and intake (Allirot et al., 2018). Participants were recruited through advertisements in social media. Included participants completed a screening assessment through an online survey system. Only baseline data was used in the present study. Complementary information about this research can be found in its main publication (Allirot et al., 2018).

Sample 4 participants were recruited from the University of Zaragoza for the psychometric validation study on the Compassion Practice Quality Scale (Navarrete et al., 2021b). Sample 5 participants took part in an ongoing study aimed to validate a short mental imagery skills training and evaluate whether it improves the quality of compassion practice. Participants of both samples underwent a compassion-based meditation and a loving-kindness meditation, respectively. All participants completed pre- and post-test assessments through an online survey system. Complementary information about the research line can be found in its main publication (Navarrete et al., 2021b).

Sample 6 participants were recruited from groups of Metacognition-Based Mindfulness and Meditation Program conducted at the University of Jaume I (Castellón, Spain) aimed at reducing stress and promoting wellbeing in the university community (Ortet et al., 2020). Participants were recruited through advertisements in social media sites and/or University communication channels. Only cross-sectional data was used for the present study (i.e., the TMS and SMS at pre-intervention). Participants were assessed through paper-and-pencil. Complementary information about this study can be found in its main publication (Ortet et al., 2020).

### 2.3. Measures

#### 2.3.1. Sociodemographic data

Data was collected on participants’ age, gender, and education level. Additionally, we collected data on previous meditation experience (yes or no question).

#### 2.3.2. Toronto mindfulness scale

The TMS (Lau et al., 2006) is a 13-item self-report measure with a 5-point response scale ranging from 0 (not at all) to 4 (very much). It assesses state mindfulness in the immediately preceding meditation practice and scores load into the curiosity (6 items; e.g., “I was curious about what I might learn about myself by taking notice of how I react to certain thoughts, feelings or sensations”) and decentering (7 items; e.g., “I experienced myself as separate from my changing thoughts and feelings”) factors. The time frame was adapted when the TMS was also administered before meditation. Higher scores (ranging from 0 to 24 scores in curiosity subscale; ranging from 0 to 28 scores of decentering subscale) indicate higher degree of state mindfulness with respect to meditation practice.

#### 2.3.3. State mindfulness scale

The SMS (Tanay and Bernstein, 2013) is a 21-item self-report measure with a 5-point Likert-type scale from 1 (not at all) to 5 (very well). It assesses state mindfulness during the previous 15 min (after meditation practice or other activity) and scores load into state mindfulness of mind (15 items; e.g., “I was aware of different emotions that arose in me”) and body (6 items; e.g., “I noticed physical sensations come and go”). In addition, a total score can be computed (ranging from 21 to 105 scores). The time frame was adapted when the SMS was also administered before meditation. Higher scores (ranging from 15 to 75 scores in mind subscale; ranging from 6 to 30 scores of body subscale) indicate greater levels of state mindfulness.

#### 2.3.4. Five facets mindfulness questionnaire

The FFMQ (Baer et al., 2006) is a 39-item self-report measure with a 5-point Liker-type scale from 1 (never or very rarely true) to 5 (very often or always true). It contains five scales of trait mindfulness: observing, describing, acting with awareness, non-judging of inner experience, and non-reactivity to inner experience. The higher the scores are, the higher the levels of trait mindfulness. The Spanish version of the original 39-item version (facets ranging from 8 to 40 scores except for non-reactivity, which ranges from 7 to 35 scores; Cebolla et al., 2012) and the 15-item version (facets ranging from 3 to 15 scores; Gu et al., 2016; Feliu-Soler et al., 2021) were used. In this sample, the scores showed adequate internal consistency with Cronbach’s alpha ranging from 0.70 to 0.87 in all facets of the FFMQ-39 and Cronbach’s alpha ranging from 0.72 to 0.85 in all facets of the FFMQ-15, except for observing (*α* = 0.56).

#### 2.3.5. The experiences questionnaire

The experiences questionnaire (EQ; Fresco et al., 2007) is a 20-item self-report measure that contains two scales: one for decentering (11 items) and one for rumination (9 items). Only the first one (EQ-Decentering) was used, in which participants rate items on a 7-point Likert-type scale from 1 (never) to 7 (all the time). A higher score indicates a higher degree of decentering (ranging from 7 to 77 scores). The Spanish version was used (Soler et al., 2014). In this sample, the EQ showed adequate internal consistency (*α* = 0.83).

#### 2.3.6. Non-attachment scale

The non-attachment scale (NAS; Sahdra et al., 2010) contains 30 items with a 6-point Liker-type scale from 1 (disagree strongly) to 6 (agree strongly) to evaluate non-attachment (e.g., “I can let go of regrets and feelings of dissatisfaction about the past”). The Spanish 7-item version was used (Feliu-Soler et al., 2016). Higher scores indicate higher non-attachment level (ranging from 7 to 42 scores). In this sample, the NAS showed adequate internal consistency (α = 0.76).

#### 2.3.7. Depression, anxiety, and stress scale

The depression, anxiety, and stress scale (DASS-21; Henry and Crawford, 2005) is a 21-item self-report measure scored on a 4-point Likert scale ranging from 0 (did not apply to me at all) to 3 (applied to me very much, or most of the time). It measures depression (7 items; e.g., “I felt that life wasn’t worthwhile”), anxiety (7 items; e.g., “I felt I was close to panic”), and stress (7 items; e.g., “I found it difficult to relax”). A total score measuring psychological distress can be calculated (ranging from 0 to 63 scores). The higher the scores are (each subscale ranging from 0 to 21 scores), the higher the levels of psychopathological symptoms will be. The Spanish version was used here (Daza et al., 2002). In this sample, the DASS-21 showed adequate internal consistency for the depression (α = 0.86), anxiety (α =0.79), and stress (α = 0.79) factors, as well as for the general distress factor (α = 0.92).

#### 2.3.8. International positive and negative affect schedule short form

The positive and negative affect schedule short form (PANAS; Thompson, 2007) is 10-item self-reporting measure that contains two 5-item Likert scales: one for positive affect and one for negative affect. It has a 5-point response format ranging from 1 (very slightly or not at all) to 5 (extremely). Higher scores indicate higher positive and negative affect (each one ranging from 5 to 25 scores). An *ad-hoc* version was used, with the items extracted from the Spanish long-version PANAS (Sandín et al., 1999). In this sample, the scores showed adequate internal consistency for the positive (α = 0.91) and negative (α = 0.73) scales.

#### 2.3.9. Forms of self-criticizing/attacking and self-reassuring scale short form

The forms of self-criticizing/attacking and self-reassuring scale short form (FSCRS-SF; Sommers-Spijkerman et al., 2018) is a self-report measure that assesses two forms of self-criticism (inadequate self and hated self) and self-reassurance (reassured self) with its 14 items being rated on a 5-point Likert scale ranging from 0 (not at all like me) to 4 (extremely like me). Higher scores indicate higher self-criticism (ranging from 0 to 20 scores in inadequacy subscale; ranging from 0 to 16 scores of self-hate subscale) and self-reassurance (ranging from 0 to 20 scores). The Spanish version was used here (Navarrete et al., 2021c). In this sample, the scores showed adequate internal consistency for the inadequate self (*α* = 0.81), hated self (*α* = 0.80), and reassured self (*α* = 0.79) scales.

Participants from Sample 1 and 2 completed the TMS, participants from samples 3, 4, and 5 answered to the SMS, and participants from Sample 6 completed both. In addition, Sample 1 completed the FFMQ-15, EQ–Decentering, NAS, and DASS-21, Sample 3 completed the FFMQ, Sample 4 completed the PANAS and FSCRS-SF and Sample 5 participants the FSCRS-SF.

### 2.4. Data analyses

Firstly, descriptive statistics (mean [M], standard deviation [SD], skewness, and kurtosis) were computed. In addition, corrected item-total correlations (rtot) were calculated for TMS and SMS items to examine how each item contributed to the overall scale. The rtot serves the purpose of identifying items that are not explicative for the assessed scale: a coefficient lower than 0.30 indicates that an item is measuring something different from the scale as a whole (DeVellis, 1991).

Then, Confirmatory Factor Analyses (CFAs) with diagonally weighted least squares (WLSMV) as the estimation method were conducted for assessing dimensionality. The minimum sample size needed was achieved considering that samples equal or above 100 participants are enough for analyzing simple models (Kline, 2015). The correlated two-factor model of the TMS (curiosity and decentering) was tested to replicate Lau et al. (2006) with the data from Sample 1. Additionally, a one-factor model with all items loading on one latent factor of state mindfulness, a bifactor model with all items loading on one general latent factor and on two uncorrelated factors, and a hierarchical two-factor model including an overarching mindfulness factor were tested. All models were calculated with and without the correlated residuals between items 5 and 6, 3 and 13, and 12 and 13 that Ireland et al. (2019) and Yu et al. (2021) proposed in their psychometric studies.

In the case of the SMS’s factorial structure, we examined the goodness-of-fit of the original structure (hierarchical two-factor model) reported by Tanay and Bernstein (2013), in addition to the following models in Samples 3, 4 and 5: a one-factor model with all items loading on one state mindfulness factor; a correlated two-factor model; and finally, a bifactor model with all items loading on one general latent factor and on two uncorrelated specific factors. All models were tested with and without the correlated residuals between items 3 and 12, 6 and 11, 10 and 16, and 15 and 16 proposed by Tanay and Bernstein (2013).

In order to test the fit of the proposed models, the following indices were calculated and interpreted using conservative and liberal cut-offs (Hu and Bentler, 1999; Schermelleh-Engel et al., 2003): the chi-square ratio (χ^2^/df) ≤ 3, the comparative fit index (CFI) and the Tucker-Lewis index (TLI) ≥ 0.95 or 0.90, the root mean square error approximation (RMSEA) ≤ 0.06 or 0.10, and the weighted root mean square residual (WRMR) ≈ 1. A practical improvement in model-fit approach was used to compare the models (difference of 0.01 or greater in TLI; Vandenberg and Lance, 2000).

Following the methodology proposed by Cuesta-Vargas et al. (2018, 2020), before assembling the TMS (Sample 1 and 2) and SMS (Samples 3–5) datasets, we tested whether the subsamples were homogeneous concerning the structure of the items represented by the covariances or correlations. Regarding the heterogeneity test for the assembled datasets, the RMSEA was chosen as the main indicator following the cut-off criteria described above (Cuesta-Vargas et al., 2018, 2020).

The internal consistency of the scales was determined by calculating Cronbach’s α, where coefficients equal to or above 0.60 indicated adequate internal consistency for exploratory research and equal to or above 0.70 for confirmatory research (Hair et al., 1998). In addition, coefficients H, omega (ω), and omega-hierarchical (ω_h_) were calculated to evaluate the reliability of the TMS and SMS from a bifactor approach (Rodriguez et al., 2016; Flora, 2020). The coefficient H measures construct replicability, with values higher than 0.80 indicating a well-defined latent variable (Hancock and Mueller, 2001). Regarding the general factor, comparing omega and omega-hierarchical indicates the reliability of the general score controlling for the specific factors. With regard to the specific factors, it provides information about their ability to reliably measure the variance by themselves controlling for the general factor. Thus, low omega-hierarchical values (<0.50) mean that the computation of specific subscale scores are not recommended (Brunner et al., 2012).

To investigate convergent/discriminant validity, Pearson correlation coefficients between TMS and FFMQ-15, EQ–Decentering, NAS, and DASS-21 were calculated with the data of Sample 1. In addition, correlations between SMS and FFMQ (Sample 3), PANAS (Sample 4), and FSCRS-SF (Samples 4 and 5) were calculated. Finally, correlations between the TMS and SMS were calculated with the data of Sample 6. The strength of the correlations was interpreted following Cohen’s guidelines (Cohen, 1988): small (*r* = 0.10–0.29), medium (*r* = 0.30–0.49), and large (*r* = 0.50–1.00).

Finally, sensitivity to change was assessed by conducting three paired-samples t-test to evaluate the impact of the 1-month Vipassana meditation retreat on the TMS scores in Sample 2, a compassion-based meditation in Sample 4 participants’ SMS scores, and a loving-kindness meditation on the SMS scores of participants from Sample 5.

Descriptive and correlation analyses and paired-samples t-test were performed with SPSS version 26. CFA was performed with Mplus version 7.4.

## 3. Results

### 3.1. Item analysis

Preliminary analyses showed that the items scores of the TMS and SMS were normally distributed, as assessed by levels of skewness and kurtosis (see Tables 2, 3). In addition, the rtot for both scales was greater than 0.30, thus suggesting adequate homogeneity of the items, except for item 4 of the TMS. However, this item was retained because the scale’s overall Cronbach’s α and McDonald’s ω were adequate.

**Table 2:** Descriptive statistics of the Toronto Mindfulness Scale and standardized factor loadings (λ) for the correlated two-factor model (+ θ_5-6_, θ_3-13_, θ_12-13_ free).

TMS items	*M (SD)*	S	K	*r_tot_*	*λ*
Factor I. Curiosity.					
3. I was curious about what I might learn about myself by taking notice of how I react to certain thoughts, feelings or sensations. [*Sentía curiosidad sobre qué podía aprender de mí mismo/a, dándome cuenta de cómo reacciono habitualmente ante ciertos pensamientos, emociones o sensaciones*.]	2.42 (1.09)	−0.58	−0.29	0.60	0.73
5. I was curious to see what my mind was up to from moment to moment. [*Sentí curiosidad por ver qué hacía mi mente momento a momento*.]	2.50 (1.10)	−0.36	−0.75	0.68	0.73
6. I was curious about each of the thoughts and feelings that I was having. [*Sentí curiosidad hacia cada uno de los pensamientos y emociones que estaba teniendo en ese momento*.]	2.39 (1.07)	−0.45	−0.53	0.68	0.70
10. I remained curious about the nature of each experience as it arose. [*Observé con curiosidad cómo era la experiencia que estaba teniendo en ese momento*.]	2.72 (0.95)	−0.51	−0.31	0.54	0.72
12. I was curious about my reactions to things. [*Sentía curiosidad por mis reacciones ante aquello que sucedía en ese momento*.]	2.37 (1.05)	−0.43	−0.58	0.67	0.74
13. I was curious about what I might learn about myself by just taking notice of what my attention gets drawn to. [*Estaba interesado/a en descubrir qué podía aprender de mí mismo/a simplemente siendo consciente de aquello que atraía mi atención en ese momento.*]	2.55 (1.08)	−0.60	−0.20	0.64	0.65
Factor II. Decentering					
1. I experienced myself as separate from my changing thoughts and feelings. [*Me percibí a mí mismo/a como algo separado de mis sentimientos y pensamientos cambiantes*.]	1.73 (1.18)	0.03	−0.91	0.32	0.46
2. I was more concerned with being open to my experiences than controlling or changing them. [*Estaba más interesado/a en estar abierto a lo que me sucedía en ese momento que en tratar de controlar o cambiar esa experiencia*.]	2.19 (0.95)	−0.10	−0.08	0.37	0.52
4. I experienced my thoughts more as events in my mind than as a necessarily accurate reflection of the way things ‘really’ are. [*Experimentaba mis pensamientos más como sucesos mentales que como un reflejo preciso de cómo son las cosas en realidad*.]	2.06 (1.07)	−0.33	−0.70	0.28	0.34
7. I was receptive to observing unpleasant thoughts and feelings without interfering with them. [*Estaba receptivo a observar mis pensamientos y sentimientos desagradables que pudieran aparecer sin necesidad de interferir en ellos*.]	2.38 (1.14)	−0.33	−0.82	0.51	0.89
8. I was more invested in just watching my experiences as they arose, than in figuring out what they could mean. [*Estaba más dedicado a simplemente observar lo que me sucedía que en interpretar su posible significado.*]	2.32 (1.13)	−0.41	−0.61	0.31	0.24
9. I approached each experience by trying to accept it, no matter whether it was pleasant or unpleasant. [*Intenté aceptar cualquier experiencia que estuviera teniendo en ese momento, sin importar si esta era agradable o desagradable*.]	2.52 (1.08)	−0.47	−0.44	0.38	0.52
11. I was aware of my thoughts and feelings without overidentifying with them. [*Era consciente de mis pensamientos y emociones sin identificarme demasiado con ellos*.]	1.95 (1.17)	0.00	−0.78	0.35	0.40

*N* = 119. TMS, Toronto Mindfulness Scale; M, mean; SD, standard deviation; S, skewness; K, kurtosis. *R*_tot_, corrected item-total correlations. Items scores range from 0 (not at all) to 4 (very much).

**Table 3:** Descriptive statistics of the State Mindfulness Scale and standardized factor loadings (λ) for the bifactor model.

SMS items	*M (SD)*	*S*	*K*	*r_tot_*	λ gen	λ spec
Factor I. State mindfulness of mind						
1. I was aware of different emotions that arose in me. [*Fui consciente de las diferentes emociones que surgieron en mí*.]	3.52 (1.08)	−0.46	−0.48	0.70	0.74	0.28
2. I tried to pay attention to pleasant and unpleasant sensations. [*Intenté atender a las sensaciones agradables y desagradables*.]	3.57 (1.18)	−0.76	−0.26	0.74	0.78	0.35
3. I found some of my experiences interesting. [*Encontré interesantes algunas de mis experiencias*.]	3.52 (1.21)	−0.58	−0.60	0.63	0.68	0.23
4. I noticed many small details of my experience. [*Me di cuenta de muchos pequeños detalles de mi experiencia*.]	3.13 (1.25)	−0.15	−1.01	0.77	0.81	0.25
5. I felt aware of what was happening inside of me. [*Fui consciente de lo que pasaba dentro de mí.*]	3.26 (1.18)	−0.33	−0.77	0.75	0.80	0.25
6. I noticed pleasant and unpleasant emotions. [*Me di cuenta de emociones agradables y desagradables*.]	3.58 (1.13)	−0.72	−0.31	0.76	0.80	0.21
7. I actively explored my experience in the moment. [*Exploré activamente mi experiencia en el momento presente*.]	3.22 (1.34)	−0.38	−1.08	0.77	0.82	0.31
10. I felt that I was experiencing the present moment fully. [*Sentí que estaba experimentando plenamente el momento presente*.]	3.06 (1.29)	−0.21	−1.09	0.77	0.81	0.17
11. I noticed pleasant and unpleasant thoughts. [*Me di cuenta de pensamientos agradables y desagradables*.]	3.53 (1.12)	−0.65	−0.32	0.76	0.84	−0.17
12. I noticed emotions come and go. [*Me di cuenta de emociones yendo y viniendo*.]	3.17 (1.22)	−0.28	−0.95	0.70	0.79	−0.31
15. I had moments when I felt alert and aware. [*Tuve momentos en los que me sentí alerta y consciente.*]	3.35 (1.20)	−0.47	−0.74	0.75	0.78	0.02
16. I felt closely connected to the present moment. [*Me sentí íntimamente conectado al momento presente*.]	3.16 (1.25)	−0.27	−0.91	0.75	0.78	0.14
17. I noticed thoughts come and go. [*Me di cuenta de pensamientos yendo y viniendo*.]	3.34 (1.14)	−0.35	−0.66	0.66	0.75	−0.48
19. I was aware of what was going on in my mind. [*Fui consciente de lo que sucedía en mi mente.*]	3.40 (1.17)	−0.48	−0.66	0.75	0.82	−0.09
20. It was interesting to see the patterns of my thinking. [*Fue interesante ver los patrones de mi pensamiento.*]	3.13 (1.30)	−0.19	−0.99	0.75	0.81	−0.07
Factor II. State mindfulness of body						
8. I clearly physically felt what was going on in my body. [*Me di cuenta claramente de lo que sucedía físicamente en mi cuerpo.*]	3.00 (1.22)	−0.15	−0.92	0.68	0.72	0.29
9. I changed my body posture and paid attention to the physical process of moving. [*Cambié la postura de mi cuerpo y presté atención al proceso físico del movimiento*.]	2.61 (1.36)	0.21	−1.28	0.55	0.60	0.23
13. I noticed various sensations caused by my surroundings (e.g., heat, coolness, the wind on my face). [*Noté varias sensaciones causadas por el entorno (p. ej.: calor, frio, el viento en mi cara).*]	3.36 (1.24)	−0.41	−0.81	0.60	0.62	0.39
14. I noticed physical sensations come and go. [*Me di cuenta de sensaciones físicas yendo y viniendo*.]	2.87 (1.17)	0.05	−0.79	0.58	0.59	0.68
18. I felt in contact with my body. [*Sentí que estaba en contacto con mi cuerpo.*]	3.05 (1.19)	−0.11	−0.88	0.76	0.81	0.14
21. I noticed some pleasant and unpleasant physical sensations. [*Me di cuenta de algunas sensaciones físicas agradables y desagradables.*]	3.31 (1.15)	−0.32	−0.79	0.67	0.74	0.07

*N* = 223. SMS, State Mindfulness Scale; M, mean; SD, standard deviation; S, skewness; *K*, Kurtosis. *R*_tot_, corrected item-total correlations. Factor loadings on general state mindfulness factor (λ gen) and specific factors (λ spec). Items scores range from 1 (not at all) to 5 (very well).

### 3.2. Dimensionality

**Figure 1:**
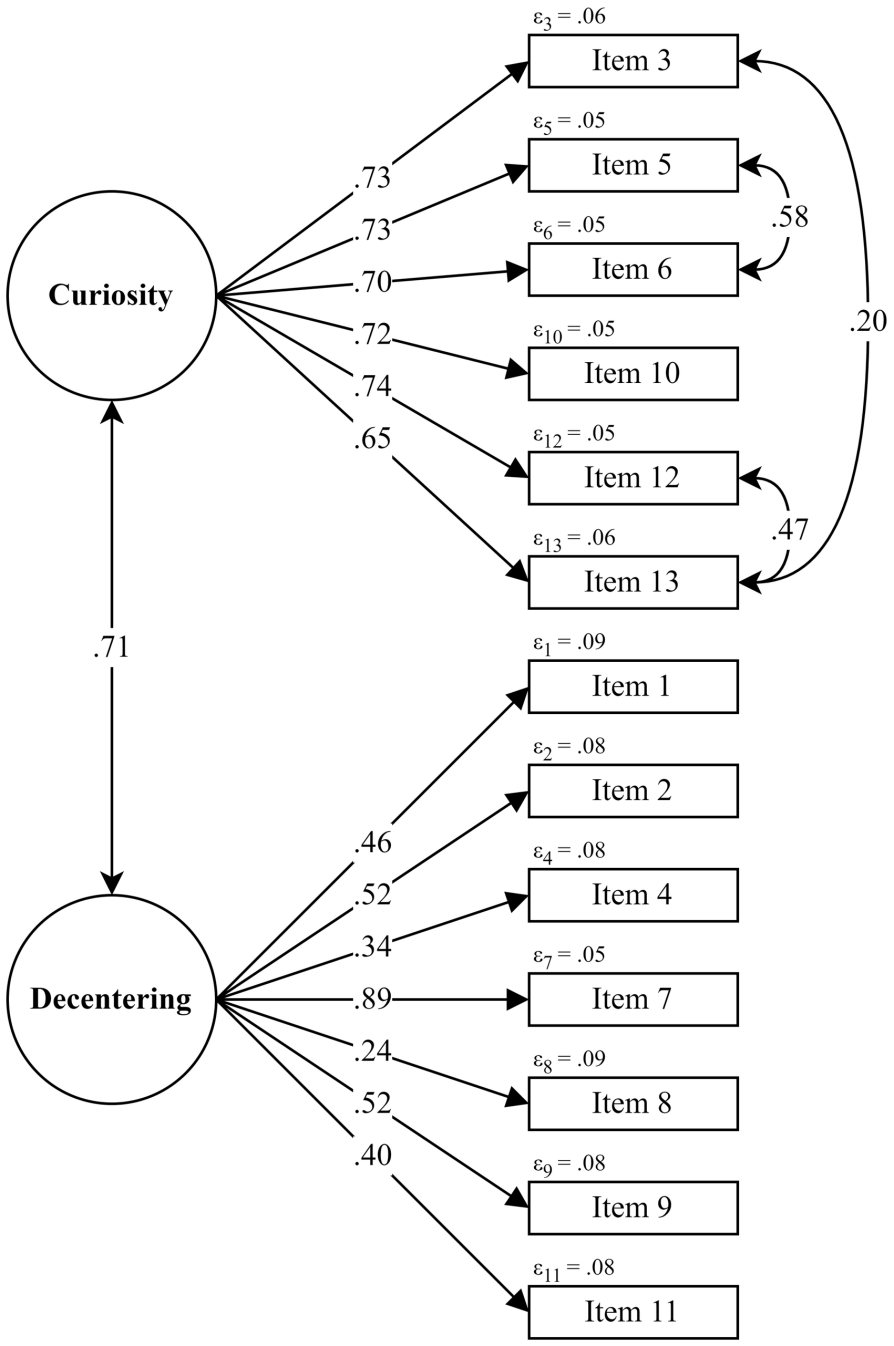
Two-factor Model of the Toronto Mindfulness Scale (TMS) obtained in the CFA.

The fit indices for the models tested in the TMS are shown in Table 4. Samples 1 and 2 were not homogeneous concerning the structure of the TMS items (RMSEA = 0.12, 90% CI [0.09–0.14]), so the CFA of the TMS was performed with data from Sample 1. The best-fitting model was the original correlated two-factor model proposed by Lau et al. (2006), though including the three pairs of correlated error terms proposed by Ireland et al. (2019) and Yu et al. (2021) (χ2/df = 2.19; *p* < 0.001; CFI = 0.93; TLI = 0.91; RMSEA = 0.10 with CI 90% [0.08, 0.12]; WRMR = 0.91). The standardized factor loadings of this two-factor model ranged from.24 (item 8) to.74 (item 12), see Figure 1 for more details. Preliminary analyses showed that the factor structures with the correlated residuals of Ireland et al. (2019) and Yu et al. (2021) separately were not supported by our data.

**Table 4:** Fit indices for the Toronto Mindfulness Scale and State Mindfulness Scale models.

Scale	Model	χ^2***^	df	CFI	TLI	RMSEA [90%CI]	WRMR
TMS (*n* = 119)	One-factor	231.38	65	0.846	0.815	0.147 [0.126, 0.167]	1.227
	One-factor (+ *θ*_5-6_, *θ*_3-13_, *θ*_12-13_ free^a^)	172.38	62	0.898	0.871	0.122 [0.101, 0.144]	1.032
	Correlated Two-factor	176.23	64	0.896	0.873	0.121 [0.100, 0.143]	1.076
	Correlated Two-factor (+ *θ*_5-6_, *θ*_3-13_, *θ*_12-13_ free^a^)	133.98	61	0.932	0.913	0.100 [0.077, 0.123]	0.908
SMS(*n* = 223)	One-factor	809.94	189	0.928	0.921	0.121 [0.113, 0.130]	1.462
	One-factor (+ *θ*_3-12_, *θ*_6-11_, *θ*_10-16,_ *θ*_15-16_ free^b^)	738.62	185	0.936	0.928	0.116 [0.107, 0.125]	1.377
	Correlated Two-factor	771.81	188	0.933	0.925	0.118 [0.109, 0.127]	1.414
	Correlated Two-factor (+ *θ*_3-12_, *θ*_6-11_, *θ*_10-16,_ *θ*_15-16_ free^b^)	705.41	184	0.940	0.931	0.113 [0.104, 0.122]	1.333
	Bifactor	564.27	168	0.954	0.943	0.103 [0.094, 0.112]	1.079
	Bifactor (+ *θ*_3-12_, *θ*_6-11_, *θ*_10-16,_ *θ*_15-16_ free^b^)	500.61	164	0.961	0.950	0.096 [0.086, 0.106]	0.993

χ^2^ (df), chi-square (degrees of freedom); CFI, Comparative Fit Index; TLI, Tucker-Lewis Index; RMSEA, root mean square error approximation; 90% CI, 90% confidence interval of the RMSEA; WRMR, weighted root means square residual; TMS, Toronto Mindfulness Scale; SMS, State Mindfulness Scale. The chosen estimator was diagonally weighted least squares (WLSMV). Indices for the TMS bifactor and the hierarchical two-factor model with and without correlated residuals models are not shown because the Mplus models did not converge. Indices for the SMS hierarchical two-factor with and without correlated residuals models are not shown because the Mplus model did not converge. ^a^Correlated residuals among items as proposed by Ireland et al. (2019) and Yu et al. (2021). ^b^Correlated residuals among items as proposed by Tanay and Bernstein (2013). ***p < 0.001.

**Figure 2:**
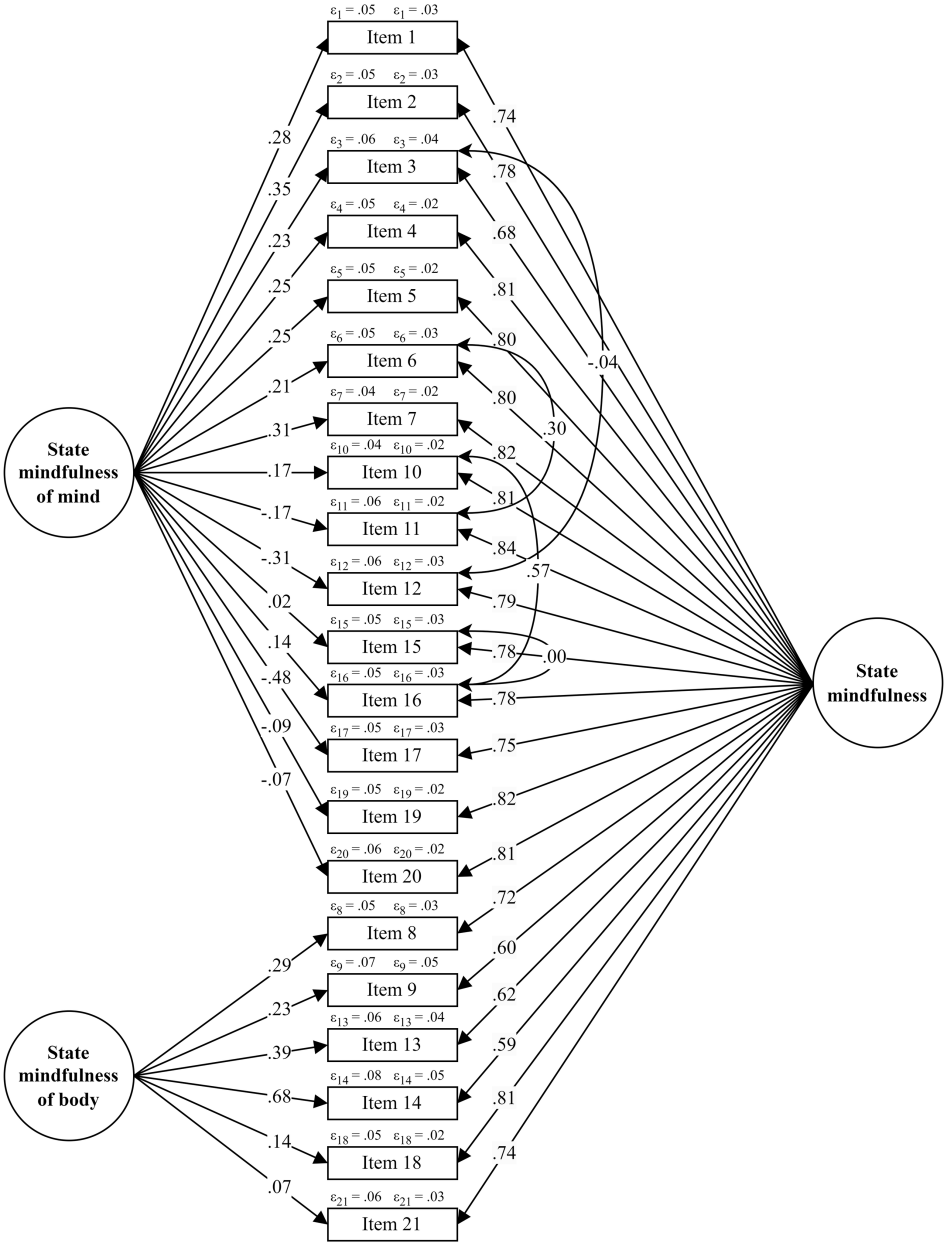
Bifactor Model of the State Mindfulness Scale (SMS) obtained in the CFA.

With regard to the SMS, when Samples 3, 4, and 5 of the dataset were tested for heterogeneity, they were found to be relatively homogeneous (RMSEA = 0.10, 90% CI [0.09–0.12]). As displayed in Table 4, the hierarchical two-factor model (with and without pairs of correlated error terms) proposed by Tanay and Bernstein (2013) was not supported. Instead, the bifactor model with the four pairs of correlated error terms suggested by the authors was the best-fitting model (χ2/df = 3.05; *p* < 0.001; CFI = 0.96; TLI = 0.95; RMSEA = 0.10 with CI 90% [0.09, 0.11]; WRMR = 0.99), showing a better fit than the bifactor solution without pairs of correlated error terms (χ^2^/df = 3.36; *p* < 0.001; CFI = 0.95; TLI = 0.94; RMSEA = 0.10 with CI 90% [0.09, 0.11]; WRMR = 1.08). The standardized factor loadings of this bifactor model ranged from.59 (item 14) to.84 (item 11), see Figure 2 for more details.

### 3.3. Reliability

Internal consistency of the TMS was adequate with Cronbach’s α and McDonald’s ω equal to or greater than.60 (αcuriosity = 0.85; αdecentering = 0.65; ωcuriosity = 0.85; ωdecentering = 0.65). Regarding the SMS, the following coefficient H, ω, and ωh values were obtained: 0.97/0.97/0.96 (SMS-total), 0.51/0.97/0.01 (SMS-mind), and.55/0.89/0.15 (SMS-body). The difference between ω and ωh for SMS-mind and SMS-body suggested that the reliability of both specific factors was very poor when controlling for the general factor of state mindfulness.

### 3.4. Convergent/discriminant validity

Relationships between the TMS subscales (curiosity and decentering) and the other questionnaires are shown in Table 5. Results showed that TMS-curiosity was positively correlated with EQ-decentering (*r* = 0.20; *p* = 0.032), being a correlation of small magnitude. TMS-curiosity was not significantly associated with FFMQ-15 facets. Moreover, TMS-decentering was positively correlated with EQ-decentering (*r* = 0.21; *p* = 0.023) and NAS-non-attachment (*r* = 0.19; *p* = 0.039). As well, TMS-decentering was correlated with FFMQ-non-reactivity (*r* = 0.28; *p* = 0.002).

**Table 5:** Convergent/discriminant validity of the Toronto Mindfulness Scale (*n* = 119).

Measure		TMS	
	*M* (SD)	Curiosity	Decentering
FFMQ-15			
Observing/noticing (3–15)	9.55 (2.84)	0.15	0.05
Describing (3–15)	9.83 (2.98)	0.11	−0.06
Acting with awareness (3–15)	9.53 (2.65)	0.00	−0.07
Non-judging of experience (3–15)	9.86 (3.06)	−0.06	−0.13
Non-reactivity to inner experience (3–15)	8.81 (2.51)	0.11	0.28**
EQ-Decentering (7–77)	34.56 (6.76)	0.20*	0.21*
NAS (7–42)	26.45 (4.84)	0.14	0.19*
DASS-21 (0–63)	20.24 (11.85)	−0.12	0.04
Depression (0–21)	5.66 (4.72)	−0.10	0.08
Anxiety (0–21)	6.10 (4.40)	−0.15	0.04
Stress (0–21)	8.45 (4.42)	−0.06	−0.02

The numbers in brackets beside subscale names is the range of possible scores. TMS, Toronto Mindfulness Scale; FFMQ-15, Five Facets Mindfulness Questionnaire (15-item version); EQ, Experiences Questionnaire – Decentering; NAS, Nonattachment scale; DASS-21, Depression Anxiety Stress Scale (21-item version). *p < 0.05; **p < 0.01.

As shown in Table 6, there was a significant association between FFMQ-observing and SMS-total (*r* = 0.35; *p* = 0.004), SMS-mind (*r* = 0.33; *p* = 0.007), and SMS-body (*r* = 0.35; *p* = 0.004). As well, SMS-body significantly correlated with FFMQ-non-reactivity. In addition, PANAS-positive affect factor was significantly associated with SMS-total (*r* = 0.44; *p* < 0.001), SMS-mind (*r* = 0.43; *p* < 0.001), and SMS-body (*r* = 0.40; *p* = 0.001). Finally, the SMS factors were negatively associated with the two factors of self-criticism, FSCRS-inadequate self (SMS-total: *r* = −0.21; *p* = 0.017; SMS-mind: *r* = −0.21; *p* = 0.017; and SMS-body: *r* = −0.18; *p* = 0.04) and FSCRS-hated self (SMS-total: *r* = −0.23; *p* = 0.010; SMS-mind: *r* = −0.21; *p* = 0.019; and SMS-body: *r* = −0.25; *p* = 0.005). In this line, there was a positive association between FSCRS-reassured self and SMS-total (*r* = 0.36; *p* < 0.001), SMS-mind (*r* = 0.35; *p* < 0.001), and SMS-body (*r* = 0.33; *p* < 0.001). Overall, the magnitude of these correlations was moderate.

**Table 6:** Convergent/discriminant validity of the State Mindfulness Scale.

Measure			SMS		
	*n*	*M* (SD)	Total	Mind	Body
FFMQ (Sample 3)					
Observing/noticing (8–40)	67	27.34 (5.18)	0.35**	0.33**	0.35**
Describing (8–40)	67	27.85 (5.09)	0.17	0.15	0.15
Acting with awareness (8–40)	64	28.41 (5.64)	0.18	0.17	0.17
Non-judging of experience (8–40)	66	29.08 (5.87)	0.06	0.01	0.15
Non-reactivity to inner experience (7–35)	67	20.73 (3.72)	0.22	0.17	0.29*
PANAS (Sample 4)					
Positive affect (5–25)	63	12.68 (4.36)	0.44**	0.43**	0.40**
Negative affect (5–25)	63	8.44 (3.23)	−0.09	−0.10	−0.07
FSCRS-SF (Samples 4 and 5)					
Inadequate self (0–20)	129	8.09 (4.55)	−0.21*	−0.21*	−0.18*
Hated self (0–16)	130	3.12 (3.59)	−0.23**	−0.21*	−0.25**
Reassured self (0–20)	130	12.58 (4.10)	0.36**	0.35**	0.33**

The numbers in brackets beside subscale names is the range of possible scores. SMS, State Mindfulness Scale; FFMQ, Five Facets Mindfulness Questionnaire (39-item version); PANAS, Positive and Negative Affect Scale; FSCRS-SF, Forms of Self-Criticizing/Attacking & Self-Reassuring Scale (Short Form). *p < 0.05; **p < 0.01.

Finally, there were statistically significant correlations between TMS-curiosity and SMS-total (*r* = 0.48; *p* = 0.001), SMS-mind (*r* = 0.48; *p* = 0.001), and SMS-body (*r* = 0.36; *p* = 0.019). As well, TMS-decentering was significantly associated with SMS-total (*r* = 0.51; *p* = 0.001), SMS-mind (*r* = 0.50; *p* = 0.001), and SMS-body (*r* = 0.40; *p* = 0.008). Overall, the magnitude of these correlations was moderate-to-large.

### 3.5. Sensitivity to change

Table 7 shows the results of the paired-samples t-tests evaluating the impact of different meditations on participants’ TMS and SMS scores. Statistically significant improvements in TMS-curiosity (*t* = −3.30, *p* = 0.003, *d* = 0.63) and TMS-decentering (*t* = −4.56, *p* < 0.001, *d* = 0.88) scores (with moderate-to-large effect sizes) were detected at the end of the 1-month Vipassana meditation retreat. Similarly, there were statistically significant changes in the SMS scores of participants who performed a compassion-based meditation (moderate-to-large effect sizes), as reflected in SMS-total (*t* = −7.10, *p* < 0.001, *d* = 0.90), SMS-mind (*t* = −7.63, *p* < 0.001, *d* = 0.97), and SMS-body (*t* = −5.07, *p* < 0.001, *d* = 0.97) scores. Regarding the participants who performed a loving-kindness meditation, significant changes in SMS scores (moderate effect sizes) from pre-meditation to post-meditation assessment were detected in the SMS-total (*t* = −7.22, *p* < 0.001, *d* = 0.70), SMS-mind (*t* = −7.13, *p* < 0.001, *d* = 0.69), and SMS-body (*t* = −8.60, *p* < 0.001, *d* = 0.67) scores.

**Table 7:** Means, standard deviations, and paired-samples *t*-test in TMS and SMS scores.

Measure	Pre-test		Post-test		*t*	*p*	Cohen’s *d* [95% CI]
	*M*	*SD*	*M*	*SD*			
1-month Vipassana meditation retreat (*n* = 22)							
TMS-Curiosity (0–24)	15.64	4.80	19.00	3.55	−3.30	0.003	0.63 [0.02, 1.23]
TMS-Decentering (0–28)	14.68	5.06	20.32	3.99	−4.56	<0.001	0.88 [0.26, 1.50]
Compassion meditation (*n* = 49)							
SMS (21–105)	55.14	19.44	74.98	14.33	−7.10	<0.001	0.90 [0.48, 1.32]
SMS-Mind (15–75)	39.31	14.27	54.35	10.26	−7.63	<0.001	0.97 [0.55, 1.39]
SMS-Body (6–30)	15.84	5.96	20.63	4.79	−5.07	<0.001	0.66 [0.25, 1.07]
Loving-kindness meditation (*n* = 84)							
SMS	68.61	17.95	82.79	13.40	−7.22	<0.001	0.70 [0.30, 1.11]
SMS-Mind	50.48	13.30	60.58	9.67	−7.13	<0.001	0.69 [0.28, 1.10]
SMS-Body	18.13	5.33	22.20	4.61	−8.60	<0.001	0.67 [0.27, 1.08]

The numbers in brackets beside subscale names is the range of possible scores. TMS, Toronto Mindfulness Scale; SMS, State Mindfulness Scale.

## 4. Discussion

In this study, we evaluated the psychometric properties of the Spanish versions of the TMS and SMS, which assess state mindfulness, in pooled non-clinical samples from the Spanish general population. Regarding Hypothesis 1, the TMS showed a correlated two-factor structure (curiosity and decentering), consistent with the original model (Lau et al., 2006), though with three pairs of correlated error terms proposed in previous psychometric studies (Ireland et al., 2019; Yu et al., 2021). So far, the correlated two-factor structure has been considered the best factorial model and previous studies has not shown evidence in favor of the presence of a general factor (Lau et al., 2006; Ireland et al., 2019; Yu et al., 2021). Regarding the SMS, a bifactor model was confirmed, instead of the hierarchical two-factor model proposed by Tanay and Bernstein (2013). Even so, both factor structures theoretically allow the scoring of the SMS subscales (mindfulness of body and mind) and a general factor of the whole scale (Brunner et al., 2012). In fact, this result might be in line with recent unpublished reports about dimensionality of the SMS by the original authors, who found support for a “similar factor structure as originally reported” (Ruimi et al., 2022, p. 13).

Regarding reliability (Hypotheses 2), the TMS scores demonstrated adequate internal consistency as expected, with similar values to those obtained in previous studies (Lau et al., 2006; Yu et al., 2021). In that sense, it should be noted that we have reported McDonald’s ω for the first time as an estimator of internal consistency of this scale. With respect to the SMS, although internal consistency analysis suggested that the total score was a reliable measure of state mindfulness, in contrast with Andrade et al. (2019), a small portion of reliably measured variance could be attributed to body and mind factors. According to our results, despite the multidimensionality of the items, these specific factors showed low reliability because they seem to be tapped primarily by the general factor of state mindfulness. The discrepancy between the present findings and those of Andrade et al. (2019) regarding the SMS might be due to the use of different approaches to model the structure of the latent variables, a hierarchical model in Andrade et al. (2019) vs. a bifactor model here. In a hierarchical model, the reliability estimates of subfactors are typically expected to be higher compared to a bifactor model because the subfactors share a substantial amount of common variance due to their direct dependence on the higher-level factor. On the contrary, the specific factors in bifactor models are intentionally designed to capture specific and unique dimensions of the construct, and they may not share as much common variance or exhibit high internal consistency (Reise et al., 2013). The presence of a strong general construct that explains much more variance than the specific constructs is a common circumstance in many self-report measures (Brunner et al., 2012; Luciano et al., 2014). Therefore, it is not recommended to compute state mindfulness of bodily sensations and state mindfulness of mind scores separately because an interpretation of a person’s level in any of both specific domains involves great uncertainty. Nevertheless, it might be useful to compute the subscales scores when complex measurement models are required (Reise et al., 2013), for instance when testing Tanay and Bernstein’s (2013) definition of state mindfulness.

With respect to convergent/discriminant validity of the TMS (Hypothesis 3), these results showed that the TMS scores were not significantly related to the facets of trait mindfulness, except for a weak association between decentering scores and non-reactivity to inner experience. Similarly, Ireland et al. (2019) found that only decentering was significantly associated to trait mindfulness (measured with the Mindful Attention Awareness Scale; Brown and Ryan, 2003). Moreover, Yu et al. (2021) reported a significant correlation between both TMS subscales scores and only observing and non-reacting. In addition, TMS scores were positively associated to decentering and non-attachment, except for curiosity with non-attachment. Similarly, Lee et al. (2010) found a significant correlation of TMS scores with decentering assessed with the EQ. However, no previous studies included the non-attachment construct, but similar ones with which curiosity and decentering (TMS) were significantly related, that is, psychological mindedness and private self-awareness (Lau et al., 2006; Yu et al., 2021). In addition, TMS scores have not shown a significant association with psychopathology symptoms in this study nor in previous ones (Lau et al., 2006; Lee et al., 2010; Yu et al., 2021), except for Ireland et al. (2019), who found a significant association between decentering and DASS-21 scores.

In this line (Hypothesis 4), SMS scores were significantly related to positive affect, but not to negative affect, similar to what Andrade et al. (2019) reported. Also, our results showed for the first time a negative association between state mindfulness and self-criticism. Meanwhile compassion research has shown interest in the influence of self-criticism on compassion states or compassion practice quality (Gilbert et al., 2006; Naismith et al., 2018; Navarrete et al., 2021b), further research is needed to study the influence of self-criticism in the process of generating mindfulness states and mindfulness meditation practice, specifically to clarify its directionality.

Our results showed a significant association between SMS scores and the observing facet of mindfulness, which Tanay and Bernstein (2013) and Andrade et al. (2019) also reported. However, there were no significant associations between SMS scores and describing non-reactivity, or non-judging meanwhile those authors did find them. It should be noted that assessing construct validity by correlating both trait and state measures might lead to counter-intuitive results (e.g., lack of or inconsistent correlations between state mindfulness and facets of trait mindfulness). However, the fact that TMS and SMS scores do not correlate very well with more stable measures (e.g., FFMQ or MAAS) probably also indicates that they are actually capturing “state” constructs. That is, state measures should show a higher correlation with other state measures than with trait measures on a given occasion (Zuckerman, 1983). In that regard, there was a significant association between the TMS and SMS scores. As previously reported, the magnitude was moderate indicating the differences in conceptual aspects of both scales (Tanay and Bernstein, 2013).

Regarding sensitivity to change (Hypothesis 5), participants who participated in the 1-month Vipassana meditation retreat, a compassion-based meditation or a loving-kindness meditation showed a significant increase in state mindfulness levels, as expected. Along this line, the TMS and SMS have been able to detect state mindfulness changes in a variety of mindfulness psychoeducation and practices (Lau et al., 2006; Tanay and Bernstein, 2013; Ruimi et al., 2022).

The main implication of this study is that the Spanish version of the TMS and SMS can be used for state mindfulness assessment in intervention research among Spanish participants from the general population. Overall, both have shown to be reliable measures with expected patterns of convergent validity and good responsiveness. However, these findings must be interpreted understanding the following limitations. First, the best-fitting CFA models achieved the standards of good model fit by the narrowest of margins. Indeed, the higher RMSEA values in the 90% confidence interval suggest that there is room for model improvements in both scales. Moreover, the variety of samples included in this study might partially bias the results because the recruitment and assessment were independent and different for each one (Podsakoff et al., 2003). In this regard, the TMS and SMS were administered either online or in person and along with different questionnaires. In addition, the study samples were recruited by a non-probability (convenience) sampling process. Therefore, it is difficult to determine how well the Spanish population is represented by them, which limits the generalizability of the findings. For instance, the samples were not representative of the Spanish general population in terms of their gender distribution (more than 70% in each sample were women) and level of education (high proportion of participants with university studies). Also, the extent to which our findings can be generalized to other Spanish-speaking countries might be limited. Moreover, all the measures used were self-report measures. Furthermore, the sample sizes were modest, especially for the TMS. Although the present samples were enough for CFA analyses according to commonly cited rules of thumb (ratio of 5:10 or a minimum sample size of 100–200; Brown, 2015), larger samples guarantee higher statistical power and robustness of the models. Regarding sensitivity to change, the studies interventions had not a control group to determine whether the changes were due to the intervention or not. Thus, the changes captured by the paired-samples t-tests could be partially (not totally) related to the meditations. Furthermore, participants were generally female, young, university educated, and without previous experience in meditation practice, which might limit the generalizability of the results to the wider population. In this line, future cross-sectional validation studies should recruit a large sample. Then, the factor structure of the TMS and the SMS Spanish versions should be tested to explore potential model modifications or alternative explanations to enhance the CFA models fit. Additionally, it is recommended to conduct replication studies using Spanish samples from various levels of education, age ranges, and in accordance with the population’s gender ratio. Finally, future research should consider studying the reliability of the SMS in detail to inform about the possibility of computing the subscale scores.

## Data availability statement

The data analyzed in this study is subject to the following licenses/restrictions: The data that support the findings of this study are available on request from the corresponding author. The data are not publicly available due to a privacy issue. Requests to access these datasets should be directed to AF-S, albert.feliu@uab.cat.

## Ethics statement

The studies involving human participants were reviewed and approved by the Ethics Committee at the Sant Joan de Déu Foundation (PIC-141-19). The patients/participants provided their written informed consent to participate in this study.

## Author contributions

AC-C, JS-M, DP, A-JS-L, JG-C, MD, JS, AC, AF-S, and JL designed and executed the study. JN and MF-M analyzed the data and wrote the manuscript. All authors reviewed and approved the final version of the manuscript for submission.

## Funding

We are grateful to the CIBER of Epidemiology and Public Health (CIBERESP CB22/02/00052; ISCIII), CIBER of Mental Health (CIBERSAM), and CIBER of Obesity and Nutrition (CIBEROBN) for their support. JN has a research contract from the Institute of Health Carlos III (ISCIII; ICI20/00080). JS-M has a PFIS predoctoral contract from the ISCIII (FI20/00034). AC-C has a FI predoctoral contract from AGAUR (FI_B/00216). AF-S acknowledges the funding from the Serra Húnter program (UAB-LE-8015). The ISCIII did not have any role in the analysis and interpretation of data, in the writing of the manuscript, or in the decision to submit the paper for publication.

## Conflict of interest

The authors declare that the research was conducted in the absence of any commercial or financial relationships that could be construed as a potential conflict of interest.

## Publisher’s note

All claims expressed in this article are solely those of the authors and do not necessarily represent those of their affiliated organizations, or those of the publisher, the editors and the reviewers. Any product that may be evaluated in this article, or claim that may be made by its manufacturer, is not guaranteed or endorsed by the publisher.
