# Supplementary material for: The Toronto Mindfulness Scale and the State Mindfulness Scale: psychometric properties of the Spanish versions
Source: Front Psychol. 2023 Jul 6;14:1212036. doi: 10.3389/fpsyg.2023.1212036 (PMC10359483; doi:10.3389/fpsyg.2023.1212036)
Supplement: Supplementary file 1 [file Table_1.DOCX]

Supplementary Material

The Toronto Mindfulness Scale and the State Mindfulness Scale: Psychometric Properties of the Spanish Versions

**Jaime Navarrete, Marta Fontana-McNally, Ariadna Colomer-Carbonell, Juan P. Sanabria-Mazo, Daniel Pinazo, Antonio-José Silvestre-López, Mark Lau, Galia Tanay, Javier García-Campayo, Marcelo Demarzo, Joaquim Soler, Ausiàs Cebolla, Albert Feliu-Soler, and Juan V. Luciano**

*** Correspondence:** Albert Feliu-Soler: [albert.feliu@uab.cat](mailto:albert.feliu@uab.cat)

# Appendix A

**Toronto Mindfulness Scale (Spanish Version)**

Estamos interesados en conocer lo que usted acaba de experimentar. A continuación, se muestra un listado de experiencias que a veces le ocurren a la gente. Por favor, lea detenidamente cada afirmación. Al lado de cada afirmación aparecen cinco opciones de respuesta: "En absoluto", "Un poco", "Moderadamente", "Bastante", y "Mucho." Por favor, marque con una cruz en qué medida está de acuerdo con cada afirmación. En otras palabras, ¿hasta qué punto la afirmación describe bien lo que usted acaba de experimentar justo ahora?

|  | **En absoluto** | **Un poco** | **Moderadamente** | **Bastante** | **Mucho** |
| --- | --- | --- | --- | --- | --- |
| 1.Me percibí a mí mismo/a como algo separado/a de mis sentimientos y pensamientos cambiantes | 0 | 1 | 2 | 3 | 4 |
| 2.Estaba más interesado/a en estar abierto a lo que me sucedía en ese momento que en tratar de controlar cambiar esa experiencia | 0 | 1 | 2 | 3 | 4 |
| 3.Sentía curiosidad sobre qué podía aprender de mí mismo/a, dándome cuenta de cómo reacciono habitualmente ante ciertos pensamientos, emociones o sensaciones | 0 | 1 | 2 | 3 | 4 |
| 4.Experimentaba mis pensamientos más como sucesos mentales que como un reflejo preciso de cómo son las cosas en realidad | 0 | 1 | 2 | 3 | 4 |
| 5.Sentí curiosidad por ver qué hacía mi mente momento a momento | 0 | 1 | 2 | 3 | 4 |
| 6.Sentí curiosidad hacia cada uno de los pensamientos y emociones que estaba teniendo en ese momento | 0 | 1 | 2 | 3 | 4 |
| 7.Estaba receptivo a observar mis pensamientos o sentimientos desagradable que pudieran aparecer sin necesidad de interferir en ellos | 0 | 1 | 2 | 3 | 4 |
| 8. Estaba más dedicado a simplemente observar lo que me sucedía que en interpretar su posible significado | 0 | 1 | 2 | 3 | 4 |
| 9.Intenté aceptar cualquier experiencia que estuviera teniendo en ese momento, sin importar si ésta era agradable o desagradable | 0 | 1 | 2 | 3 | 4 |
| 10. Observé con curiosidad cómo era la experiencia que estaba teniendo en ese momento | 0 | 1 | 2 | 3 | 4 |
| 11.Era consciente de mis pensamientos y emociones sin identificarme demasiado con ellos | 0 | 1 | 2 | 3 | 4 |
| 12.Sentía curiosidad por mis reacciones ante aquello que sucedía en ese momento | 0 | 1 | 2 | 3 | 4 |
| 13. Estaba interesado/a en descubrir qué podía aprender de mí mismo/a simplemente siendo consciente de aquello que atraía mi atención en ese momento | 0 | 1 | 2 | 3 | 4 |

**Scoring**

**Curiosity**

ITEM_3 + ITEM_5 + ITEM_6 + ITEM_10 + ITEM_12 + ITEM_13

**Decentering**

ITEM_1 + ITEM_2 + ITEM_4 + ITEM_7 + ITEM_8 + ITEM_9 + ITEM_11

**State Mindfulness Scale (Spanish Version)**

A continuación, se presenta un listado de frases. Por favor, utiliza la escala de respuestas para indicar si las frases describen bien tus experiencias en los últimos 15 minutos.

1= en absoluto

2= un poco

3= algo

4= bien

5= muy bien

| 1. Fui consciente de las diferentes emociones que surgieron en mí. | **1** | **2** | **3** | **4** | **5** |
| --- | --- | --- | --- | --- | --- |
| 1. Intenté atender a las sensaciones agradables y desagradables. | **1** | **2** | **3** | **4** | **5** |
| 1. Encontré interesantes algunas de mis experiencias. | **1** | **2** | **3** | **4** | **5** |
| 1. Me di cuenta de muchos pequeños detalles de mi experiencia. | **1** | **2** | **3** | **4** | **5** |
| 1. Fui consciente de lo que pasaba dentro de mí. | **1** | **2** | **3** | **4** | **5** |
| 1. Me di cuenta de emociones agradables y desagradables. | **1** | **2** | **3** | **4** | **5** |
| 1. Exploré activamente mi experiencia en el momento presente. | **1** | **2** | **3** | **4** | **5** |
| 1. Me di cuenta claramente de lo que sucedía físicamente en mi cuerpo. | **1** | **2** | **3** | **4** | **5** |
| 1. Cambié la postura de mi cuerpo y presté atención al proceso físico del movimiento. | **1** | **2** | **3** | **4** | **5** |
| 1. Sentí que estaba experimentando plenamente el momento presente. | **1** | **2** | **3** | **4** | **5** |
| 1. Me di cuenta de pensamientos agradables y desagradables. | **1** | **2** | **3** | **4** | **5** |
| 1. Me di cuenta de emociones yendo y viniendo. | **1** | **2** | **3** | **4** | **5** |
| 1. Noté varias sensaciones causadas por el entorno (ej: calor, frio, el viento en mi cara). | **1** | **2** | **3** | **4** | **5** |
| 1. Me di cuenta de sensaciones físicas yendo y viniendo. | **1** | **2** | **3** | **4** | **5** |
| 1. Tuve momentos en los que me sentí alerta y consciente. | **1** | **2** | **3** | **4** | **5** |
| 1. Me sentí íntimamente conectado al momento presente. | **1** | **2** | **3** | **4** | **5** |
| 1. Me di cuenta de pensamientos yendo y viniendo. | **1** | **2** | **3** | **4** | **5** |
| 1. Sentí que estaba en contacto con mi cuerpo. | **1** | **2** | **3** | **4** | **5** |
| 1. Fui consciente de lo que sucedía en mi mente. | **1** | **2** | **3** | **4** | **5** |
| 1. Fue interesante ver los patrones de mi pensamiento. | **1** | **2** | **3** | **4** | **5** |
| 1. Me di cuenta de algunas sensaciones físicas agradables y desagradables. | **1** | **2** | **3** | **4** | **5** |

**Scoring**

**SMS (mind factor)**

ITEM_1 + ITEM_2 + ITEM_3 + ITEM_4 + ITEM_5 + ITEM_6 + ITEM_7 + ITEM_10 + ITEM_11 + ITEM_12 + ITEM_15 + ITEM_16 + ITEM_17 + ITEM_19 + ITEM_20

**SMS (body factor)**

ITEM_8 + ITEM_9 + ITEM_13 + ITEM_14 + ITEM_18 + ITEM_21

**SMS (total score)**

SMS (mind factor) + SMS (body factor)
